# Supplementary material for: Cell-lineage controlled epigenetic regulation in glioblastoma stem cells determines functionally distinct subgroups and predicts patient survival
Source: Nat Commun. 2022 Apr 25;13:2236. doi: 10.1038/s41467-022-29912-2 (PMC9038925; doi:10.1038/s41467-022-29912-2)
Supplement: Supplementary file 1 — Supplementary Information [file 41467_2022_29912_MOESM1_ESM.pdf]

Supplementary information:

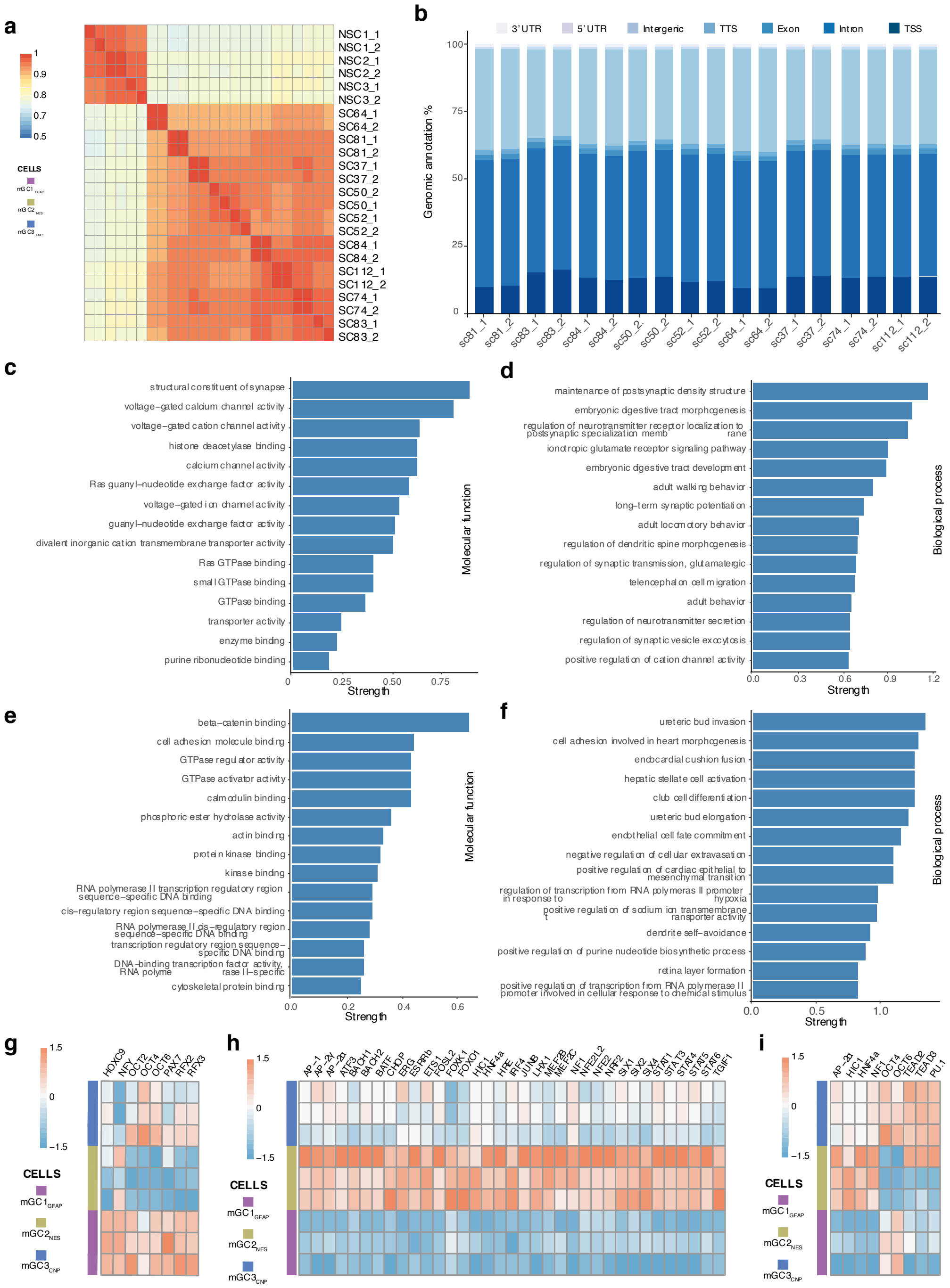

Supplementary Fig. 1. Analyses of ATAC-seq data from mouse GSC cultures of different origin.

(**a**) Pearson correlation matrix of ATAC-seq libraries of mouse NSC and GSC duplicate samples. (**b**) Genomic annotation of all ATAC peaks in duplicate mouse GSC samples. (**c-f**) Gene ontology enrichment analyses of genes annotated to unique ATAC peaks of the $mGC1_{GFAP}$ (**c, d**) and $mGC2_{NES}$ (**e, f**) groups, respectively. (**g-i**) Heatmaps of significantly enriched TF motifs in different origin of mouse GSC cultures.

Source data are provided as a Source Data file for Supplementary Fig. 1a-i.

a

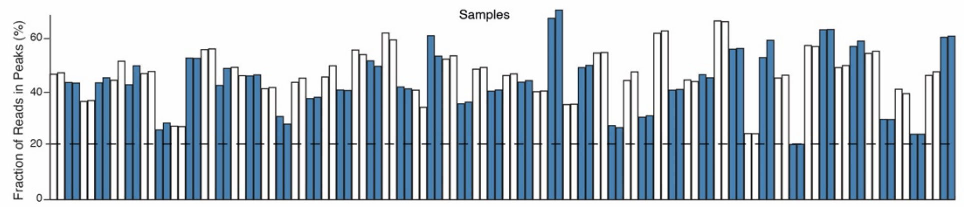

b

Sample	U3009	U3042	U3263	U3468	U3129	U3180	U3123	U3047	U3056	U3164	U3017	U3461	U3323	U3440	U3169
R	0.9140	0.8414	0.9191	0.8960	0.9417	0.9683	0.9526	0.9346	0.9185	0.9414	0.9301	0.9566	0.9748	0.9517	0.9773
Sample	U3053	U3020	U3230	U3065	U3118	U3117	U3085	U3002	U3005	U3028	U3039	U3093	U3062	U3068	U3046
R	0.9595	0.9501	0.9781	0.9768	0.9746	0.9690	0.9692	0.9759	0.9637	0.9489	0.9448	0.9226	0.9903	0.9707	0.9363
Sample	U3086	U3155	U3112	U3172	U3429	U3071	U3073	U3024	U3027	U3013	U3078	U3033	U3308	U3198	U3121
R	0.9708	0.9585	0.8916	0.9286	0.9409	0.8937	0.9244	0.8277	0.8290	0.9277	0.9170	0.9378	0.9340	0.9147	0.9502
Sample	U3137	U3060	U3202	U3110	U3008	U3173	U3140	U3084	U3031	U3082	U3037	U3034	U3035	U3167	U3088
R	0.9703	0.9613	0.9815	0.9754	0.9479	0.9754	0.9578	0.9719	0.9491	0.8544	0.9670	0.9252	0.9829	0.9685	0.9647

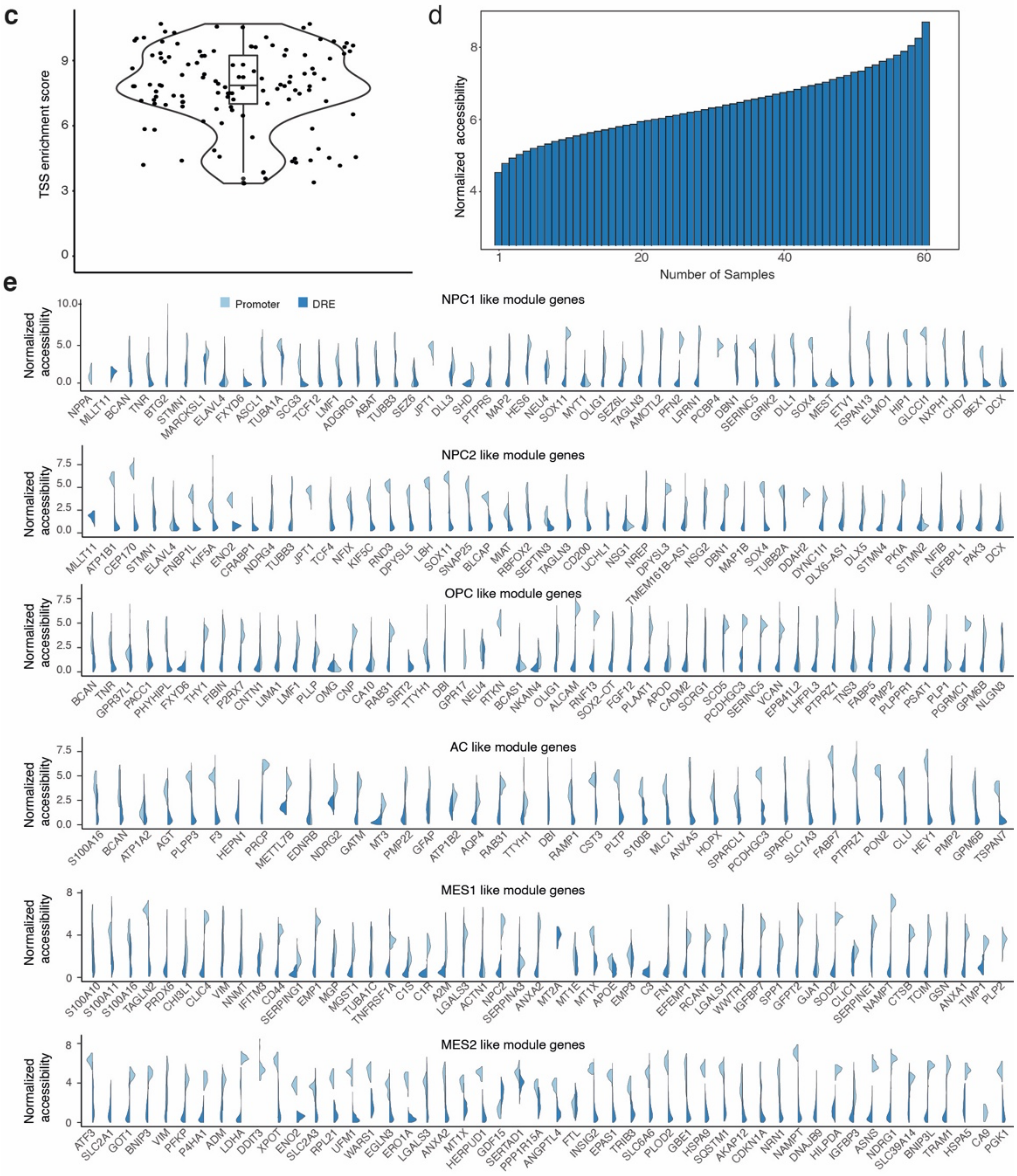

Supplementary Fig. 2. Analyses of ATAC-seq data from 60 patient-derived GSC cultures. (**a**) Fraction of reads in peaks (FRiP) in duplicate samples of hGSC cultures. Dashed line mark 20% FRiP. (**b**) Pearson correlation analysis of duplicate hGSC ATAC-seq samples. *R* = Pearson correlation. (**c**) TSS enrichment score of ATAC-seq data for each hGSC culture.

sample size (n = 120) for the box plot. The box plot in the violin plot shows the minima (bottom dot), the maxima (top dot), the median (middle line) and the first and third quartiles (boxes), whereas the whiskers show 1.5× the interquartile range IQR above and below the box.
(**d**) Normalized chromatin accessibility of region-unique ATAC peaks in different numbers of hGSC cultures. (**e**) Violin plots of cohort-wide distribution of chromatin accessibility at promoter and distal regulatory element (DRE) regions of GSC meta module genes.
Source data are provided as a Source Data file for Supplementary Fig. 2a-e.

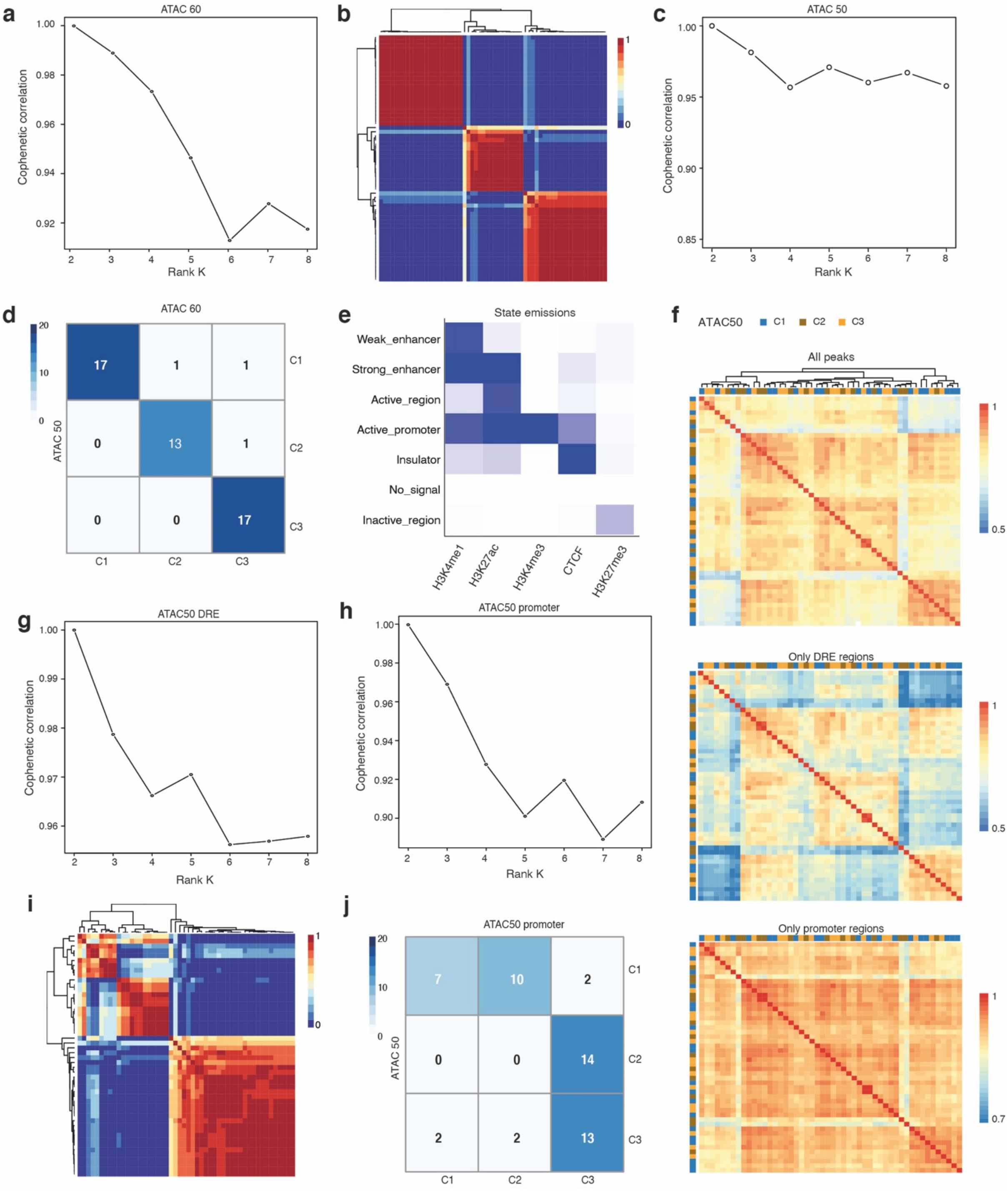

Supplementary Fig. 3. Cluster analysis of hGSC ATAC-seq data.

(**a**) Cophenetic correlation coefficient curve for cluster analysis of 60 hGSC ATAC-seq samples. (**b**) Non-negative matrix factorization (NMF) clustering of ATAC-seq data from 60 hGSC cultures. (**c**) Cophenetic correlation coefficient curve for cluster analysis of 50 hGSCs ATAC-seq samples. (**d**) Overlap of ATAC60 clusters with ATAC50 clusters. (**e**) Definitions of the seven chromatin-states used in the chromatin state discovery and characterization (ChromHMM) software. (**f**) Pearson correlation heatmaps of all ATAC peaks (top), ATAC peaks of DRE regions (middle) and ATAC peaks of promoter regions (bottom). Samples are arranged in the same order in all heatmaps, based on Pearson correlation of all ATAC peaks. (**g**) Cophenetic correlation coefficient curve for cluster analysis of chromatin accessibility of promoter regions in ATAC-seq data from 50 hGSC cultures. (**h**) Cophenetic correlation

coefficient curve for cluster analysis of chromatin accessibility of DRE regions in ATAC-seq data from 50 hGSC cultures. (**i**) NMF clustering of ATAC-seq data from promoter regions of 50 hGSC cultures. (**j**) Overlap of ATAC50 clusters with ATAC50 promoter clusters.
Source data are provided as a Source Data file for Supplementary Fig. 3a-j.

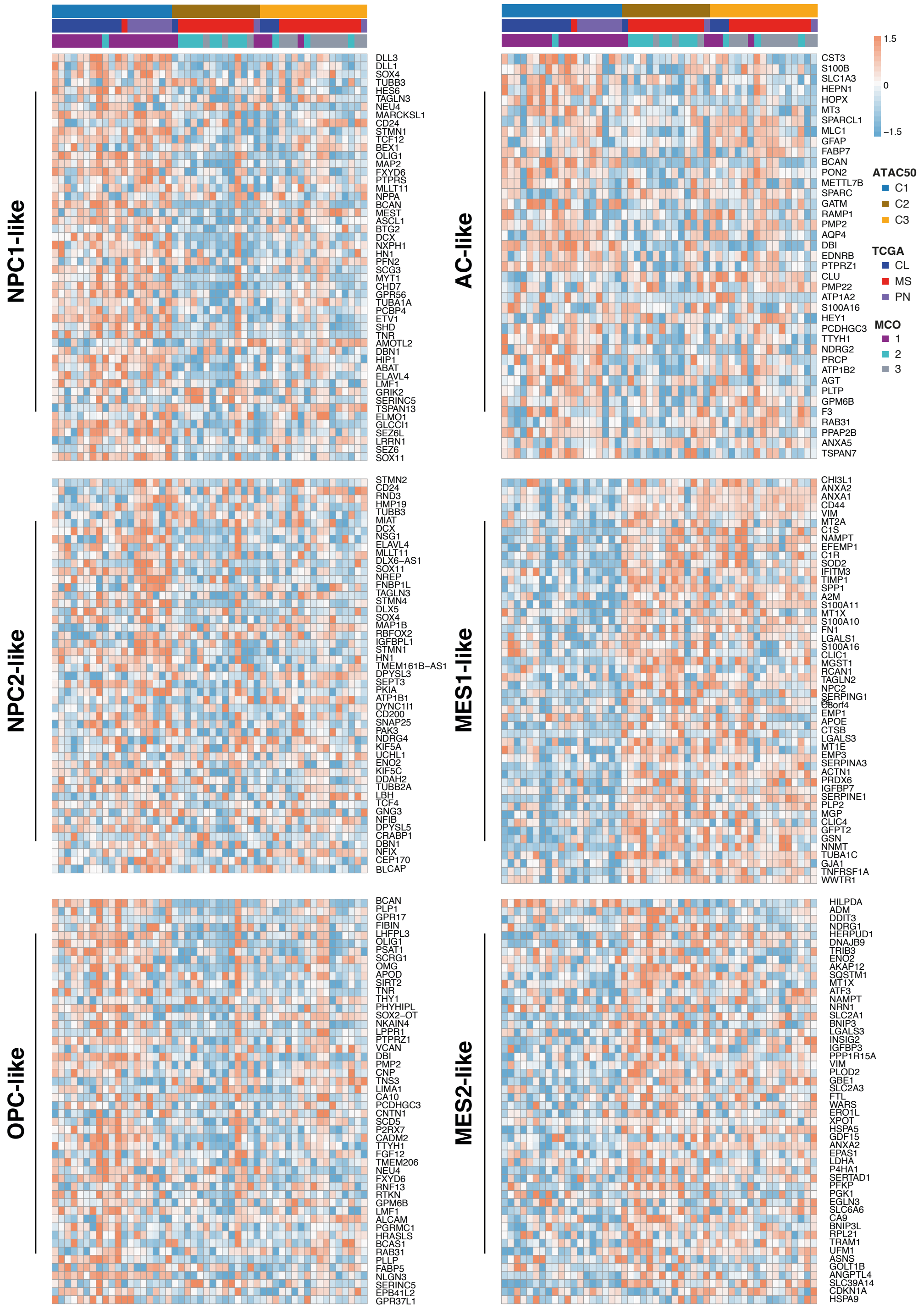

Supplementary Fig. 4. Gene expression of GSC meta module genes in 50 hGSC cultures. Source data are provided as a Source Data file for Supplementary Fig. 4.

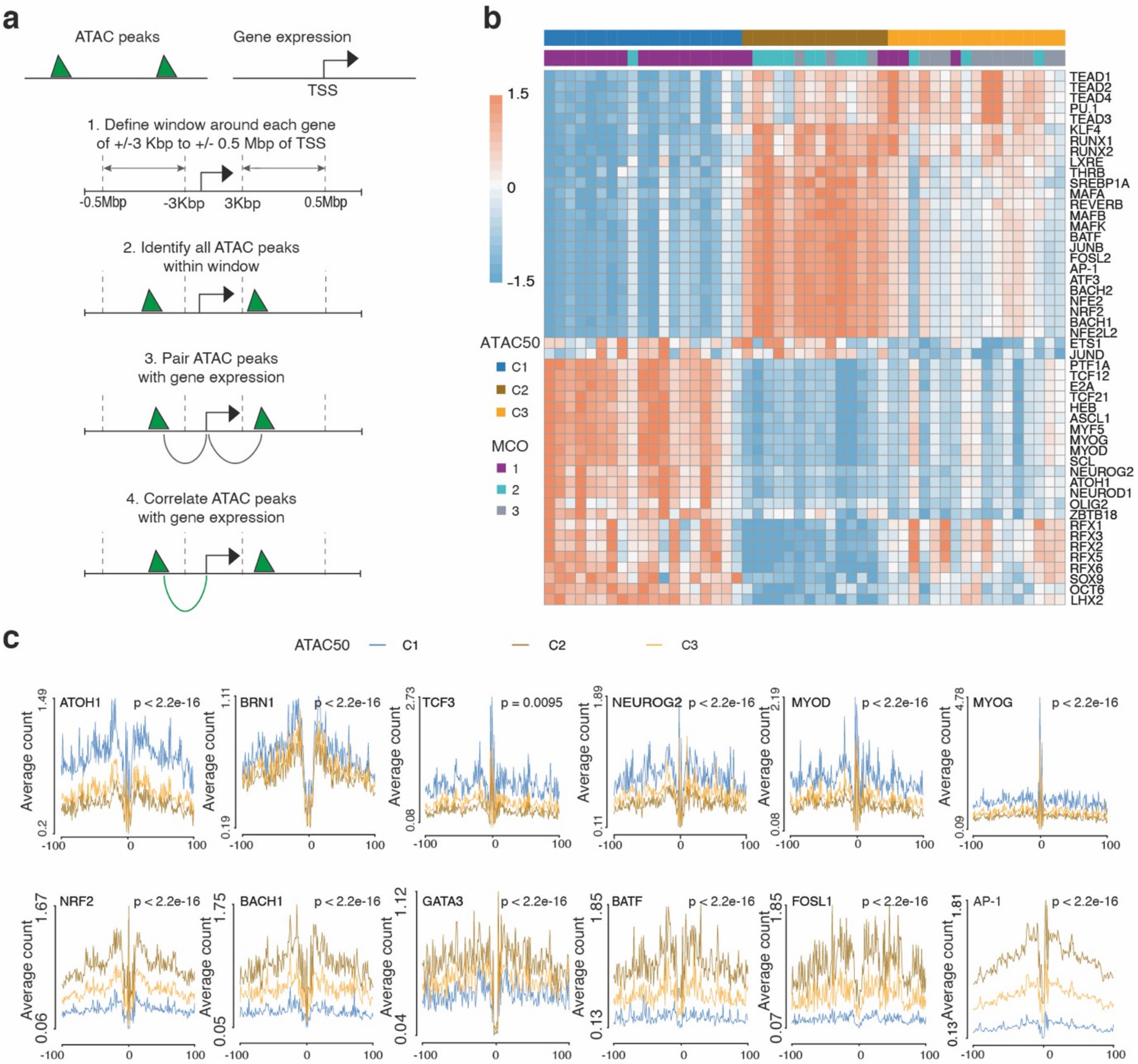

Supplementary Fig. 5. Molecular characterization of ATAC50 clusters.
(**a**) Overview of the ATAC peak-to-gene linking prediction analysis. (**b**) Top-50 most significantly enriched and variable TF motifs in ATAC50 cluster-specific ATAC peaks. (**c**) TF footprint analysis of cluster-specific ATAC50 peaks. Friedman-Nemenyi test was performed, and p-values are labeled.
Source data are provided as a Source Data file for Supplementary Fig. 5b, 5c.

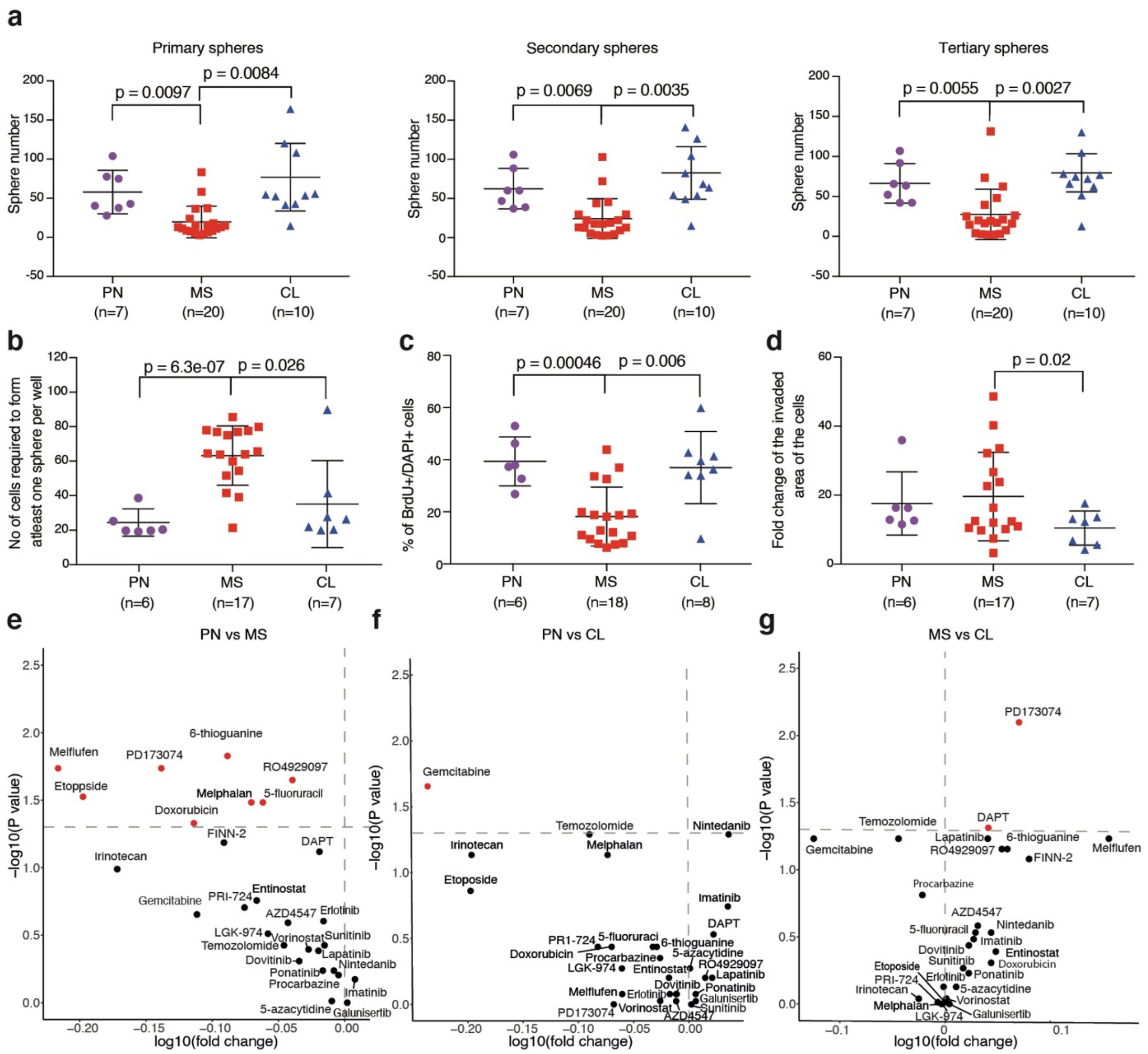

Supplementary Fig. 6. Functional analyses of hGSC cultures grouped by TCGA subtypes.

(**a**) Consecutive sphere assays. Data show mean ± SEM. Two-sided Welch's t-test was performed on all pair-wise comparisons, significant differences are indicated. n, number of cell cultures. (**b**) ELDA. Data show mean ± SEM. Two-sided Welch's t-test was used. n, number of cell cultures. (**c**) Frequency of BrdU positive cells. Data show mean ± SEM. Two-sided Welch's t-test was performed on all pair-wise comparisons, significant differences are indicated. n, number of different cell cultures analyzed. (**d**) Collagen invasion assay measuring the invaded area at 24 hours. Data show mean ± SEM. Two-sided Welch's t-test was performed on all pair-wise comparisons, significant differences are indicated. n, number of cell cultures. (**e-g**) Volcano plots of pair-wise comparisons of AUC scores in ATAC50 C1 (n=12), C2 (n=7) and C3 (n=10) cultures to 28 anti-cancer drugs. Red circles indicate significantly different drug response. n, number of cell cultures. Two-sided Mann-Whitney test was performed on all pair-wise comparisons without multiple adjustment. (**e**) ATAC50 PN versus MS. Red circles in upper left corner, PN more sensitive. (**f**) ATAC50 PN versus CL. Red circle in upper left corner, PN more sensitive. (**g**) ATAC50 MS versus CL. Red circles in upper right corner, CL more sensitive.

Source data are provided as a Source Data file for Supplementary Fig. 6a-g.

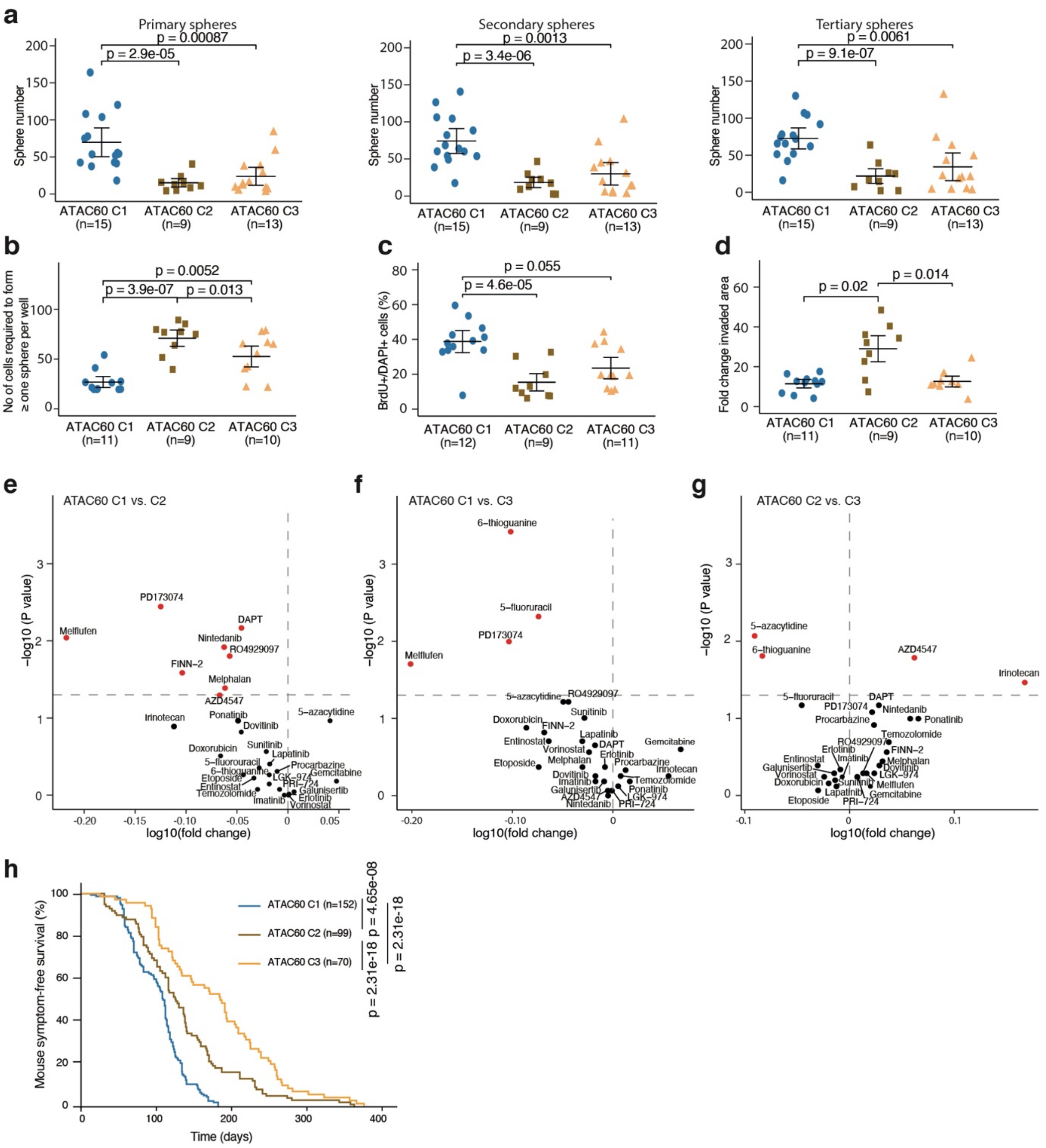

Supplementary Fig. 7. Functional analyses of hGSC cultures grouped by ATAC60.
(**a**) Consecutive sphere assays comparing ATAC60 clusters. Data show mean ± SEM. Two-sided Welch's t-test was performed on all pair-wise comparisons, significant differences are labeled. n, number of cell cultures. (**b**) ELDA comparing ATAC60 clusters. Data show mean ± SEM. Two-sided Welch's t-test was used, and significant differences are labeled with p-value. n, number of cell cultures. (**c**) Frequency of BrdU positive cells. Data show mean ± SEM. Two-sided Welch's t-test was performed on all pair-wise comparisons, significant differences are labeled with p-value. (**d**) Collagen invasion assay measuring the invaded area at 24 hours. Data show mean ± SEM. Two-sided Welch's t-test was performed on all pair-wise comparisons, significant differences are labeled with p-value. n, number of cell cultures. (**e-g**) Volcano plots of pair-wise comparisons of AUC scores in ATAC60 C1 (n=11), C2 (n=8) and C3 (n=10) cultures to 28 anti-cancer drugs. Red circles indicate significantly different drug response. n, number of cell cultures. Two-sided Mann-Whitney test was performed on all pair-wise comparisons without multiple adjustment. (**e**) ATAC60 C1 versus C2. Red circles in upper left corner, C1 more sensitive. (**f**) ATAC60 C1 versus C3. Red circles in upper left corner, C1

more sensitive. (**g**) ATAC60 C2 versus C3. Red circles in upper left corner, C2 more sensitive. Red circles in upper right corner, C3 more sensitive. (**h**) Kaplan-Meier analysis of symptom-free survival of immune-deficient mice intracranially injected with 10^4, 10^5 or $2x10^5$ cells from 34 different hGSC cultures. Cultures are divided based on ATAC60 clusters, C1 (n=12), C2 (n=12), C3 (n=10). Log-rank (Mantel-Cox) test was used, and p-value is labeled, n, number of injected mice.
Source data are provided as a Source Data file for Supplementary Fig. 7a-h.

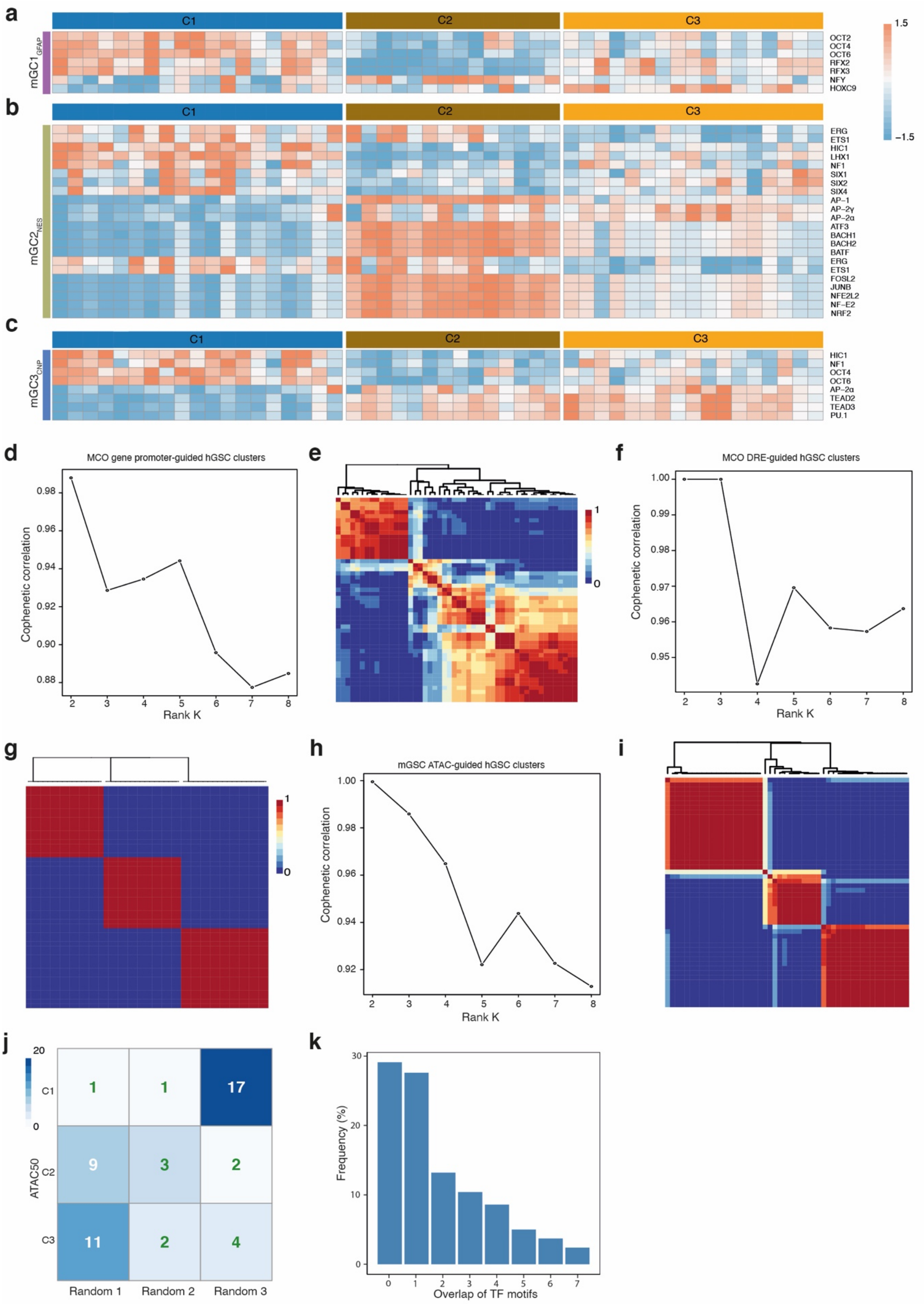

Supplementary Fig. 8. Cross-species analyses using mouse GSC data to produce clusters of hGSC cultures.

(**a-c**) Correlation analysis of significantly enriched and variable TF motifs of unique mouse and human ATAC peaks. TF motifs of each mGSC group, mGC1$_{GFAP}$ (**a**), mGC2$_{NES}$ (**b**),

$mGC3_{CNP}$ (**c**), were compared to the complete hGSC TF motif list, and all TFs with significant positive correlation to at least one mGSC group are shown in the heatmaps. (**d**) Cophenetic correlation coefficient curve for cluster analysis of ATAC peaks of promoter regions of 166 human MCO homologue genes. (**e**) Non-negative matrix factorization (NMF) clustering of chromatin accessibility data of promoter regions of 166 human MCO homologue genes. (**f**) Cophenetic correlation coefficient curve for cluster analysis of ATAC peaks of DRE regions of 166 human MCO homologue genes. (**g**) NMF clustering of chromatin accessibility data of DRE regions of 166 human MCO homologue genes. (**h**) Cophenetic correlation coefficient curve for cluster analysis of differential mGSC ATAC peak-guided hGSC ATAC peaks. (**i**) NMF clustering of chromatin accessibility data of mGSC ATAC peak guided homologous hGSC ATAC peaks. (**j**) Overlap of ATAC50 clusters with the average clusters of 1000 times NMF analysis of 4834 randomly selected ATAC peaks. (**k**) Frequency of top-10 enriched overlapping TF motifs in a 1000 times repeated comparison of 2075 randomly selected mGSC ATAC peaks with their corresponding human homologue ATAC peaks.
Source data are provided as a Source Data file for Supplementary Fig. 8a-k.
