# Supplementary material for: Cell-lineage controlled epigenetic regulation in glioblastoma stem cells determines functionally distinct subgroups and predicts patient survival
Source: Nat Commun. 2022 Apr 25;13:2236. doi: 10.1038/s41467-022-29912-2 (PMC9038925; doi:10.1038/s41467-022-29912-2)
Supplement: Supplementary file 3 — Description of Additional Supplementary Files [file 41467_2022_29912_MOESM3_ESM.pdf]

Supplementary Figures 1-8 are in the separated file.
Source data is in the separated file.
Supplementary Data 1-9 are in separate files.

Supplementary Data 1. Mouse and human cell cultures used in the study.

Supplementary Data 2. Differential mouse GSC ATAC peaks and genes annotated to these peaks.

Supplementary Data 3. Significantly enriched and variable TF motifs of differential mouse GSC ATAC peaks.

Supplementary Data 4. Differential human GSC ATAC50 peaks and genes annotated to these peaks.

Supplementary Data 5. DRE ATAC peak-to-gene linkage prediction.

Supplementary Data 6. Significantly enriched and variable TF motifs of differential human GSC ATAC peaks.

Supplementary Data 7. Significantly enriched ATAC50 cluster-unique TF motifs.

Supplementary Data 8. AUC values of drug response analyses of human GSC cultures.

Supplementary Data 9. Survival of immune-deficient mice orthotopically transplanted with human GSCs.
