# Supplementary material for: Cell-lineage controlled epigenetic regulation in glioblastoma stem cells determines functionally distinct subgroups and predicts patient survival
Source: Nat Commun. 2022 Apr 25;13:2236. doi: 10.1038/s41467-022-29912-2 (PMC9038925; doi:10.1038/s41467-022-29912-2)
Supplement: Supplementary file 13 — Reporting Summary [file 41467_2022_29912_MOESM13_ESM.pdf]

nature research

Corresponding author(s): Xingqi Chen & Lene Uhrbom

Last updated by author(s): April 7, 2022

Reporting Summary

Nature Research wishes to improve the reproducibility of the work that we publish. This form provides structure for consistency and transparency in reporting. For further information on Nature Research policies, see our Editorial Policies and the Editorial Policy Checklist.

Statistics

For all statistical analyses, confirm that the following items are present in the figure legend, table legend, main text, or Methods section.

n/a	Confirmed	
☐	☒	The exact sample size (*n*) for each experimental group/condition, given as a discrete number and unit of measurement
☐	☒	A statement on whether measurements were taken from distinct samples or whether the same sample was measured repeatedly
☐	☒	The statistical test(s) used AND whether they are one- or two-sided<br>*Only common tests should be described solely by name; describe more complex techniques in the Methods section.*
☒	☐	A description of all covariates tested
☐	☒	A description of any assumptions or corrections, such as tests of normality and adjustment for multiple comparisons
☐	☒	A full description of the statistical parameters including central tendency (e.g. means) or other basic estimates (e.g. regression coefficient) AND variation (e.g. standard deviation) or associated estimates of uncertainty (e.g. confidence intervals)
☐	☒	For null hypothesis testing, the test statistic (e.g. *F*, *t*, *r*) with confidence intervals, effect sizes, degrees of freedom and *P* value noted<br>*Give P values as exact values whenever suitable.*
☒	☐	For Bayesian analysis, information on the choice of priors and Markov chain Monte Carlo settings
☒	☐	For hierarchical and complex designs, identification of the appropriate level for tests and full reporting of outcomes
☐	☒	Estimates of effect sizes (e.g. Cohen's *d*, Pearson's *r*), indicating how they were calculated

Our web collection on statistics for biologists contains articles on many of the points above.

Software and code

Policy information about availability of computer code

Data collection	Illumina NovaSeq 6000 was used to perform sequencing.
Data analysis	All detailed scripts used in this study were deposited in following link:<br>https://github.com/chenlab2019/GSC;<br>https://zenodo.org/record/6374965#.Yjl5AS8w2u4<br>Bowtie2 (version v2.4.5, http://bowtie-bio.sourceforge.net/bowtie2/index.shtml) was used to align ATAC-seq reads to hg19 genome.<br>Picard tools version 1.119 (https://broadinstitute.github.io/picard/) was used to remove duplicate reads.<br>MACS2 (version 2.2.7.1, https://hbctraining.github.io/Intro-to-ChIPseq/lessons/05_peak_calling_macs.html)was used to perform peak calling.<br>NMF algorithm was used to build NMF clusters.<br>chomVAR (https://github.com/GreenleafLab/chromVAR)were used to perform TF analysis.<br>Footprint analysis was performed by HINT-ATAC (https://www.regulatory-genomics.org/hint/introduction/).<br>Experimental assay analysis was performed using R version 3.5.3.<br>Drug response analysis were done in R version 3.5.3.<br>samtools 1.9 software (http://www.htslib.org/);<br>bedtools v2.29.2 software (https://github.com/arq5x/bedtools2);<br>ggplot2 v3.3.5 package (https://cran.r-project.org/web/packages/ggplot2/index.html);<br>homer v4.11 software (http://homer.ucsd.edu/homer/motif/);

For manuscripts utilizing custom algorithms or software that are central to the research but not yet described in published literature, software must be made available to editors and reviewers. We strongly encourage code deposition in a community repository (e.g. GitHub). See the Nature Research guidelines for submitting code & software for further information.

Data

Policy information about availability of data

All manuscripts must include a data availability statement. This statement should provide the following information, where applicable:

- Accession codes, unique identifiers, or web links for publicly available datasets
- A list of figures that have associated raw data
- A description of any restrictions on data availability

All raw data of ATAC-seq from human GSC and mouse GSC generated in this study have been deposited in Gene Expression Omnibus of National Center for Biotechnology information under access code GSE163853[https://www.ncbi.nlm.nih.gov/geo/query/acc.cgi?acc=GSE163853]. Figure 1-4, Figure 7 and Supplementary Figure 1-6 are associated with these raw data.
The publicly available gene expression data of human GSC used in this study is from previous published report and available in the Gene Expression Omnibus of National Center for Biotechnology information under access code GSE91393 [https://www.ncbi.nlm.nih.gov/geo/query/acc.cgi?acc=GSE91393]. Figure 4b is associated with this gene expression data.
The in vivo survival data of intracranially injected immune-deficient mice from previous published reports is available in Supplementary data 9. Figure 6a and Figure 6b are associated with these data.
The remaining data are available within the Article, Supplementary Information or Source Data file. Source data is provided with this paper.

Field-specific reporting

Please select the one below that is the best fit for your research. If you are not sure, read the appropriate sections before making your selection.

☒ Life sciences ☐ Behavioural & social sciences ☐ Ecological, evolutionary & environmental sciences

For a reference copy of the document with all sections, see nature.com/documents/nr-reporting-summary-flat.pdf

Life sciences study design

All studies must disclose on these points even when the disclosure is negative.

Sample size	Sequencing experiments were performed in duplicates for each biological sample. For mouse glioblastoma stem cell lines, there were three biological samples for each cell of origin (These number cover different mouse cell origin of glioblastoma). For human glioblastoma stem cell lines, no sample-size calculation was performed. a saturation curve analysis was performed to estimate the number of samples needed to cover all the potential chromatin accessibility regions. No sample-size calculation was performed.
Data exclusions	For human glioblastoma stem cell lines, we had analyzed a few IDH-mutant samples that were excluded in the manuscript since we wanted to focus on IDH-wildtype samples. For all computational analysis, sequencing data with low quality (Pearson correlation < 0.8) was excluded. This was done based on a pre-established cutoff. The exclusion criteria is set based on the good reproducibility, and will not affect our conclusion.
Replication	Sphere formation, proliferation, extreme limiting dilution and invasion assays for selected cell lines were repeated three times. All our experiments were successful.
Randomization	Mouse and human GSC clusters were built using NMF algorithm with cophenetic value as selected parameters. The group is without biased-selection.
Blinding	Blinding is not necessary during sample collection and library preparation because the basic sequencing analysis was done with an automated pipeline. Additional analyses were performed in an unbiased manner following the established criteria from software instruction.

Reporting for specific materials, systems and methods

We require information from authors about some types of materials, experimental systems and methods used in many studies. Here, indicate whether each material, system or method listed is relevant to your study. If you are not sure if a list item applies to your research, read the appropriate section before selecting a response.

Materials & experimental systems

n/a	Involved in the study
[ ]	[x] Antibodies
[ ]	[x] Eukaryotic cell lines
[x]	[ ] Palaeontology and archaeology
[ ]	[x] Animals and other organisms
[x]	[ ] Human research participants
[x]	[ ] Clinical data
[x]	[ ] Dual use research of concern

Methods

n/a	Involved in the study
[x]	[ ] ChIP-seq
[x]	[ ] Flow cytometry
[x]	[ ] MRI-based neuroimaging

Antibodies

Antibodies used

Rat monoclonal anti-BrdU (1:100, Abcam, Ab6326)
Goat Anti-Rat Alexa 555 (1:400, Invitrogen, A21434)

Validation

Antibodies are commercially available and have been validated by the following manufacturers Abcam and Invitrogen.
https://www.abcam.com/brdu-antibody-bu175-icr1-proliferation-marker-ab6326.html
https://www.thermofisher.com/antibody/product/Goat-anti-Rat-IgG-H-L-Cross-Adsorbed-Secondary-Antibody-Polyclonal/A-21434

Eukaryotic cell lines

Policy information about cell lines

Cell line source(s)

All cell cultures have been published and were established locally in accordance with ethical approvals (Uppsala ethical review board 2007/353 for human samples and the local animal ethics committee C237/12 and C182/14 for mouse samples). Mouse GSC and NSC cultures were established from primary mouse glioblastoma tissues. Human GSC cultures were established from patient-derived glioblastoma tissue samples.

Authentication

The majority of human cell lines have been authenticated by STR profiling.

Mycoplasma contamination

All cell lines have been tested regularly for mycoplasma infection using a PCR-based method and the KAPA kit, and have been negative.

Commonly misidentified lines
(See ICLAC register)

No cross-contaminations have been reported to ICLAC for the cell lines used in this study.

Animals and other organisms

Policy information about studies involving animals; ARRIVE guidelines recommended for reporting animal research

Laboratory animals

NOD.CB17-Prkdcscid/NCrHsd NOD-SCID mice (Harlan) of both sexes

Wild animals

Study did not involve wild animals.

Field-collected samples

Study did not involve field-collected samples

Ethics oversight

All procedures in mice were performed in accordance with the rules and regulations of Uppsala University and after approval from the local animal ethics committee.

Note that full information on the approval of the study protocol must also be provided in the manuscript.
